# Translation Initiation Factor eIF4E Positively Modulates Conidiogenesis, Appressorium Formation, Host Invasion and Stress Homeostasis in the Filamentous Fungi *Magnaporthe oryzae*

**DOI:** 10.3389/fpls.2021.646343

**Published:** 2021-06-16

**Authors:** Wajjiha Batool, Ammarah Shabbir, Lili Lin, Xiaomin Chen, Qiuli An, Xiongjie He, Shu Pan, Shuzun Chen, Qinghe Chen, Zonghua Wang, Justice Norvienyeku

**Affiliations:** ^1^Fujian University Key Laboratory for Plant-Microbe Interaction, College of Plant Protection, Fujian Agriculture and Forestry University, Fuzhou, China; ^2^State Key Laboratory for Ecological Pest Control of Fujian and Taiwan Crops, The School of Life Sciences, Fujian Agriculture and Forestry University, Fuzhou, China; ^3^Key Laboratory of Green Prevention and Control of Tropical Plant Diseases and Pests, Ministry of Education, College of Plant Protection, Hainan University, Haikou, China; ^4^Institute of Oceanography, Minjiang University, Fuzhou, China

**Keywords:** *Magnaporthe oryzae*, eIF4E3, translational regulation, fungal pathogenesis, mRNA

## Abstract

Translation initiation factor eIF4E generally mediates the recognition of the 5’cap structure of mRNA during the recruitment of the ribosomes to capped mRNA. Although the eIF4E has been shown to regulate stress response in *Schizosaccharomyces pombe* positively, there is no direct experimental evidence for the contributions of eIF4E to both physiological and pathogenic development of filamentous fungi. We generated *Magnaporthe oryzae eIF4E* (*MoeIF4E3*) gene deletion strains using homologous recombination strategies. Phenotypic and biochemical analyses of *MoeIF4E3* defective strains showed that the deletion of *MoeIF4E3* triggered a significant reduction in growth and conidiogenesis. We also showed that disruption of *MoeIF4E3* partially impaired conidia germination, appressorium integrity and attenuated the pathogenicity of Δ*Moeif4e3* strains. In summary, this study provides experimental insights into the contributions of the eIF4E3 to the development of filamentous fungi. Additionally, these observations underscored the need for a comprehensive evaluation of the translational regulatory machinery in phytopathogenic fungi during pathogen-host interaction progression.

## Introduction

Transcriptional and translational regulation of gene expression plays a central role in cell differentiation, physiological development and regulates cellular processes that enable living organisms to readily adapt to harsh or changing environmental conditions (Thach, 1992). Translation initiation events play a fundamental but crucial role in gene expression by regulating the assemblage of the cellular machinery required to transform mRNAs to protein (translation). In eukaryotes, a set of proteins known as translation initiation factors (eIFs) usually mediate the translational initiation process during different cellular and environmental stress (Lacerda et al., 2017). The eIF4F complex comprises three subunits (eIF4A, eIF4E, and eIF4G) that mediate the translational regulation of mRNAs in a cap-dependent manner and selectively regulate the translation of a large proportion of mRNAs by modulating the binding of the 43S pre-initiation complex to mRNAs (Aitken and Lorsch, 2012).

The eIF4E of the eIF4F complex activates the translational initiation process by encasing the 5′cap (methylated Guanine) of eukaryotic mRNA. The eIF4E binds the 5′cap of mRNA to trigger the folding-back of the mRNA’s poly-A tail to form a loop with the 5′cap to facilitate the correct positioning of the ribosome at mRNA and to prevent the premature initiation of the translation process (Hinnebusch and Lorsch, 2012). In mammals, eIF4E subunit remains inactive by forming a complex with one of its binding proteins (4E-BP1); the subsequent phosphorylation of eIF4E stimulates their release and creates an eIF4G binding site (Montero et al., 2019). Whereas in *Saccharomyces cerevisiae*, functional homologs of 4E-BPs proteins p20 (Altmann et al., 1997) and Eap1 (Cosentino et al., 2000) have been identified that competes with eIF4G for binding to eIF4E. Thus, to regulate the activity of eIF4E, eukaryotic translation initiation factor 4E-binding protein (4E-BP) and eIF4G are considered as key proteins in cap-dependent translation (Gallagher et al., 2019).

Previous studies have shown that initiation factors play an essential role in the biosynthesis of virulence proteins during host-pathogen interaction. Most pathogenic microbes suppress the biosynthesis of defense and stress-responsive proteins in host organisms by targeting and compromising the functionality of translational initiation factors in the host cells. For example, eIF5A is involved in the induction of cell death in *A. thaliana* during *Pseudomonas syringae* infection (Hopkins et al., 2008). Whereas eIF4E in *Capsicum sp.* (Ibiza et al., 2010), *Cucumis melo* (Nieto et al., 2006), *Hordeum vulgare* (Stein et al., 2005), *Oryza sativa* (Boisnard et al., 2007), and *Pisum sativum* (Bruun-Rasmussen et al., 2007), act as a potential candidate to provide resistance during plant-virus interactions. In *Schizosaccharomyces pombe*, eIF4E2 showed upregulation under different stress conditions (Ptushkina et al., 2004). These observations highlight the role of eIF4E2 in regulating cellular stress response in eukaryotes. Although translation initiation factors, including eIF4E2, have been implicated in biotic stress responses, the direct or indirect contribution of eIF4E to the pathogenesis of filamentous fungi are still unknown.

Fungal diseases are significant obstacles to attaining optimal yields in agricultural practice and thus threaten global food security (Skamnioti and Gurr, 2009; Fones et al., 2017). *Magnaporthe oryzae* (rice blast fungus), a causal agent of and the most destructive disease of rice worldwide (Talbot, 2003) has been reported as a new emergence in the form of wheat-blast during the past few years (Islam et al., 2016; Inoue et al., 2017). Infections caused by this intractable pathogen lead to the annual destruction of enough rice to feed more than 60 million people (Scheuermann et al., 2012). One million hectares of annual crop loss due to rice blast disease has just been reported in China alone (Khush and Jena, 2009). Through extensive genetic studies over the past few decades, several genes were reported to have a critical role in the morphological and infection cycle of *M. oryzae*. However, eIF4E role has not yet been reported to have a role in the plant pathogenic fungi, *M. oryzae*. Here, we identified and deployed cell biology, functional genetic, and biochemical techniques to functionally evaluate the contributions of eukaryotic translation initiation factor eIF4E isoform (MoeIF4E3) to morphological and pathogenic development of the cosmopolitan and economically destructive rice blast fungus pathogen (Talbot, 2003; Norvienyeku et al., 2020).

## Results

### Identification of eIF4E Gene in *M. oryzae*

To identify putative eIF4E domain-containing proteins in *M. oryzae*, amino acids (aa) sequences of eIF4E domain-containing protein from *Botrytis cinerea*, *Fusarium graminearum*, and *Neurospora crassa* were used to run blastP and reverse blastP searches in the publicly available fungi and oomycete genomic resource database^1^ and Kyoto Encyclopedia for Genes and Genomes (KEGG)^2^. Blast search analysis identified a putative eIF4E protein (MoeIF4E3) encoded by a gene with the open reading frame MGG_08170, whereas results obtained from the KEGG database assisted blast analysis identified two more *M. oryzae* eIF4E proteins MoeIF4E and MoeIF4E1 along with MoeIF4E3. However, protein sequence similarity index recorded for MoeIF4E and MoeIF4E1 comparative to eIF4E3 sequences identified from *B. cinerea*, *F. graminearum*, and *N. crassa* has lower than 40% sequence similarity while MoeIF4E3 recorded sequence similarity index of ∼60%, indicating that *M. oryzae* most likely exist as a single copy, *MoeIF4E3* (MGG_08170). Further class level orthologous analyses, interestingly, showed that apart from the classes *Leotiomycetes, Sordariomycetes*, and some members of *Eurotiomycetes*, fungi, and Oomycetes from other taxonomic groups lacks eIF4E3 orthologues (Supplementary Table 1). Likewise, eIF4E3 from *Saccharomyces cerevisiae*, *Arabidopsis thaliana*, *Drosophila melanogaster*, and *Homo Sapiens* show less than 40% sequence similarity to MoeIF4E3 (Supplementary Table 2 and Supplementary Figure 1), and thus were not included for further study.

**FIGURE 1:**
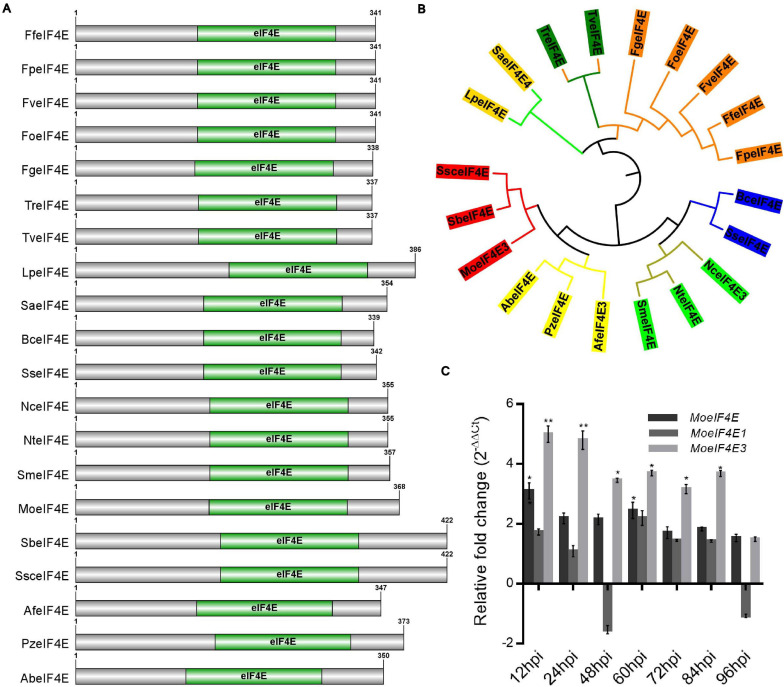
Domain architecture and phylogeny of *M. oryzae* eIF4E3 containing proteins. **(A)** Domain architecture of different fungal proteins containing eIF4E3 domains. **(B)** Shows maximum likelihood phylogeny constructed entirely for eIF4E3 domain sequence (amino acid) across different fungal species (Lp: *Lomentospora prolificans*, Sa: *Scedosporium apiospermum*, Tr: *Trichoderma reesei*, Tv: *Trichoderma virens*, Fg: *Fusarium graminearum*, Fo: *Fusarium oxysporum*, Fv: *Fusarium verticillioides*, Ff: *Fusarium fujikuroi*, Fp: *Fusarium proliferatum*, Bc: *Botrytis cinerea*, Ss: *Sclerotinia sclerotiorum*, Nc: *Neurospora crassa*, Nt: *Neurospora tetrasperma*, Sm: *Sordaria macrospora*, Af: *Aspergillus fumigatus*, Pz: *Penicilliopsis zonata*, Ab: *Aspergillus brasiliensis*, Mo: *Magnaporthe oryzae*, Sb: *Sporothrix brasiliensis*, Ssc: *Sporothrix schenckii*. **(C)** The expression level of *MoeIF4E*, *MoeIF4E1*, and *MoeIF4E3* genes during different stages of host-pathogen interaction. Vegetative hyphae were used as the control stage and were assumed as unity (the expression level of *MoeIF4E*, *MoeIF4E1*, and *MoeIF4E3* at hyphal stage = 1). (*) and (**) represent significant differences *p* < 0.05 and *p* < 0.01 respectively.

Additional eIF4E domain sequence homology and phylogenetic analyses were carried out using the Pfam domain prediction web server revealed that all the protein sequences (Supplementary Table 2) obtained from the fungal species selected for the phylogenetic analysis possess a conserved eIF4E domain (Figure 1A). Results obtained from phylogenetic analyses conducted in this study using domain sequences showed that MoeIF4E3 shared a more recent phylogenetic lineage with *Sporothrix brasiliensis* and *Sporothrix schenckii* (Figure 1B). The class-level absence and within-class divergence of eIF4E3 domain proteins suggest that organisms, especially members in the class *Sordariomycetes*, possibly acquired the eIF4E3 domain-containing proteins as an adaptation mechanism during evolution. Furthermore, assessing the expression level of *MoeIF4E*, *MoeIF4E1*, and *MoeIF4E3* at different stages of infection using qPCR showed a general up-regulation in the expression profile for *MoeIF4E3* at all stages of *M. oryzae* infection compared to *MoeIF4E* and *MoeIF4E1* (Figure 1C). These results indicate that MoeIF4E, MoeIF4E1, and MoeIF4E3 isomers likely exert differential influence on the infectious development of *M. oryzae*.

### Generation of Single *MoeIF4E3* Gene Deletion Strains

**FIGURE 2:**
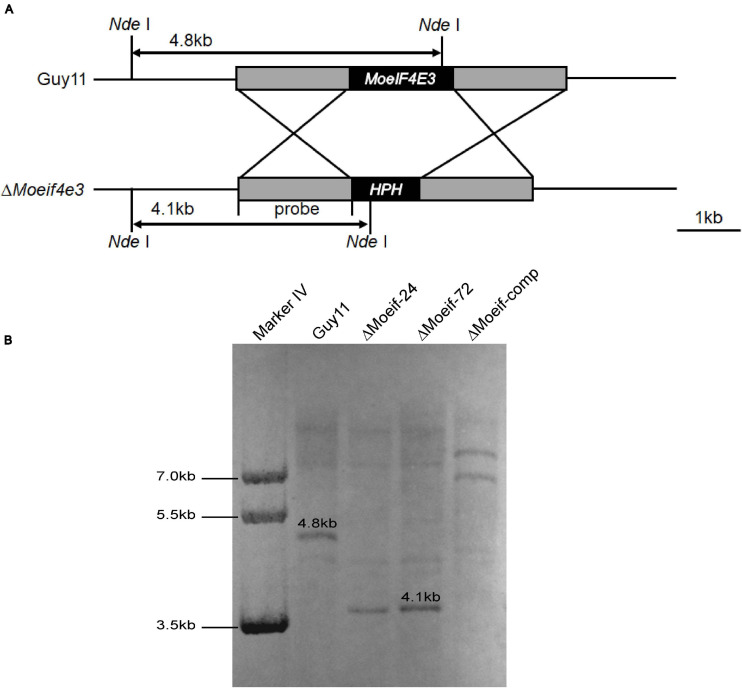
Targeted gene deletion of *MoeIF4E3* in *M. oryzae*. **(A)** Schematic presentation of targeted disruption of *MoeIF4E3* using homologs recombination approach. **(B)** Southern blot results showing successful replacement of *MoeIF4E3* by a single insertion of Hygromycin phosphotransferase (hph) ORF at *MoeIF4E3* loci.

As *MoeIF4E3* expression was higher during host-pathogen interaction than for other potential MoeIF4E isomers, we investigated the influence of *MoeIF4E3* on the physiological and infectious development of the rice blast fungus. We used the homologous recombination targeted gene deletion technique and successfully generated *MoeIF4E3* gene deletion strains (Δ*Moeif-24*, Δ*Moeif-72*). Potential other transformants were prescreened by PCR assays using gene-specific primer pairs (Supplementary Table 3) and later confirmed with qPCR (Supplementary Figure 1) and Southern-blotting (Figure 2). Results obtained from these confirmation assays identified two independent *MoeIF4E3* gene deletion strains (Δ*Moeif-24*, Δ*Moeif-72*) in which the *MoeIF4E3* ORF region was successfully replaced with Hygromycin phosphotransferase (hph) gene inserted in the ORF.

### Sub-Cellular Localization of eIF4E3 Identified in *M. oryzae*

**FIGURE 3:**
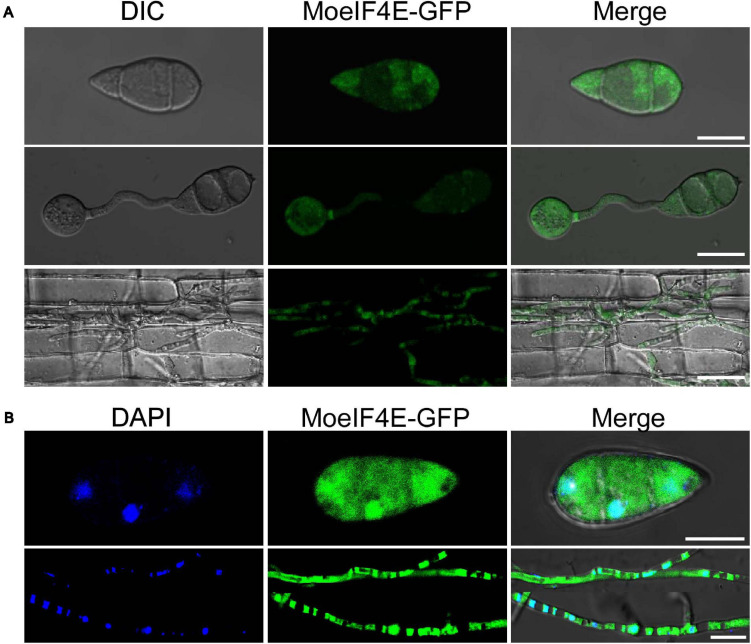
Subcellular localization of MoeIF4E3 in *M. oryzae* and *in planta*. **(A)** The localization pattern of MoeIF4E3 in asexual spore, during spore germination and in invasive hyphae during plant infection. **(B)** Localization pattern of MoeIF4E3 with DAPI staining in asexual spore and invasive hyphae. Localization of MoeIF4E3-GFP was visualized using Nikon laser confocal microscopy. The scale bar is 20 and 40 μm.

The eIF4E is an essential component of eukaryotic translation initiation machinery that functions within the nucleus and cytoplasm of eukaryotes. Previous studies in mammals showed that eIF4E localizes to the protoplasm (Willett et al., 2006; Osborne and Borden, 2015). Contrasting results from other independent experiments in *S. pombe* suggest the identified eIF4E inherently localizes to the cytosolic pool and across the organelle membrane periphery (Ptushkina et al., 2004). Therefore, to confirm the subcellular localization of MoeIF4E3 in *M. oryzae*, we constructed MoeIF4E3-GFP fused complementation vectors containing neomycin-resistant cassettes under the *MoeIF4E3* native promoter and transformed the contras into the protoplast prepared from the Guy11 strain. The transformants were selected on culture media supplemented with neomycin, prescreened by PCR using a listed primer pair (Supplementary Table 3). Successful transformants harboring the MoeIF4E3-GFP fusing construct were subsequently subjected to confocal microscopy. We observed that MoeIF4E3-GFP localizes to the protoplasm of *M. oryzae*. Further assessment of the localization of MoeIF4E3-GFP during host-pathogen interaction showed that MoeIF4E3-GFP retained the protoplasmic localization during the development of *M. oryzae* in the host plant (Figures 3A,B). These results support the association of MoeIF4E3 with the protein translation machinery in the cytoplasm and nucleus.

### MoeIF4E3 Deletion Results in Developmental Defects of *M. oryzae*

**FIGURE 4:**
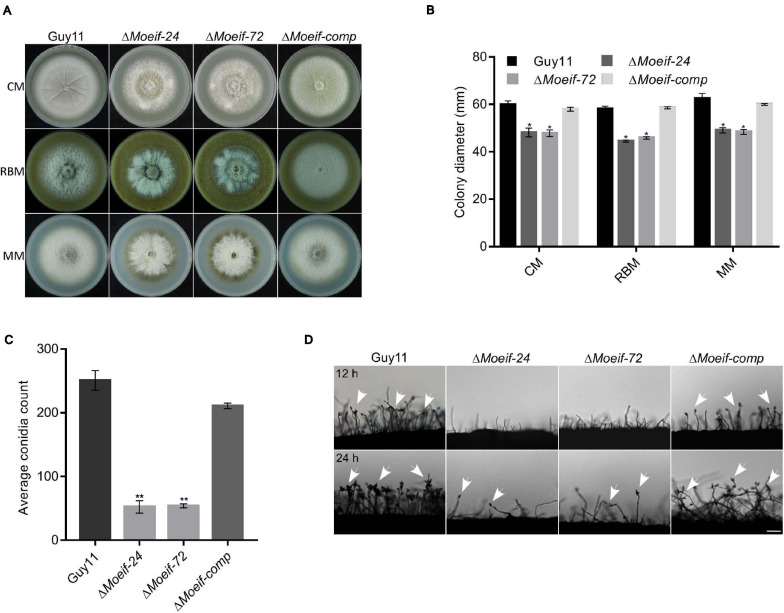
MoeIF4E3 contributes significantly to the vegetative growth and conidiogenesis of *M. oryzae.* **(A)** Depicts the average colony diameter and morphology of Δ*MoeIF4E3* mutants (Δ*Moeif-24*, Δ*Moeif-72*), complemented strain Δ*Moeif-comp* and the wild-type Guy11 strain cultured on CM, RBM, and MM for 10 days. **(B)** Statistical demonstration of the average growth rate of respective mutant strains and the wild-type Guy11 on CM, RBM, and MM for 10 days. **(C)** A statistical representation of conidiation capacity of *MoeIF4E3* gene deletion strains and their complement relative to the Guy11 wild-type strain. **(D)** The reduction in conidiophore and conidia-bearing capabilities of conidiophores of the strains on rice bran medium. The statistical data were analyzed with a Microsoft Excel spreadsheet and GraphPad-prism7. Error bars represent the standard deviation from at least three independent replicates, and (*) and (**) represent significant differences (*p* < 0.05 and *p* < 0.01, respectively) between the wild type Guy11 and the respective knockout mutants according to ordinary one-way ANOVA. Bar, 10 μm.

To ascertain roles for MoeIF4E3 in the morphological development of rice blast fungus, we measured colony diameter of the Δ*Moeif-24*, Δ*Moeif-72*, complementation (*Moeif-comp*), and wild-type strains cultured on CM media for 10-days. Comparative analyses of growth records showed that targeted gene deletion of *MoeIF4E3* triggered a significant reduction (≈ 30%) in the radial growth of the Δ*Moeif4e3* strains. Similar vegetative growth defects were observed during the growth of the Δ*Moeif4e3* strains on minimum media (MM) and rice bran media (RBM) (Figures 4A,B). These results showed that MoeIF4E3 directly or indirectly promotes radial growth of the blast fungus. Given the observed increase in aerial hyphae production in the Δ*Moeif4e3* strains, we alternatively postulated that MoeIF4E3 likely positively regulates radial growth and negatively regulates aerial morphogenesis in hyphae in *M. oryzae.*

Spores play a crucial role in facilitating the survival and the efficient dissemination of fungi under harsh environmental conditions and disease perpetuation (Wyatt et al., 2013). In an attempt to establish the role of MoeIF4E3 in the asexual reproduction of *M. oryzae*, the Δ*Moeif-24*, Δ*Moeif-72*, complementation, and the wild type strains were cultured on rice bran media for 10-days. The vegetative hyphae were scratched off, and the plates were exposed to a continuous white fluorescent light to induce sporulation. Spores from the individual strains were washed and collected to make a spore suspension, to be counted under a light microscope. Results obtained from conidia count assays showed that compared to the wild type, targeted gene deletion of MoeIF4E3 triggered an approximately 70% reduction in the number of conidia produced by the Δ*Moeif4e3* mutants (Figure 4C) compared to the wild type control. From these observations, we conclude that MoeIF4E3 contributes positively to conidiogenesis in *M. oryzae.*

We assessed essential sporulation parameters, including; conidiation onset, the spatial distribution of conidiophores, and the numbers of conidia per conidiophore, to unravel how MoeIF4E3 likely regulates conidiogenesis. These investigations showed that the deletion of *MoeIF4E3* compromised conidiophorogenesis and drastically reduced the number of conidia per conidiophore (Figure 4D). We infer that MoeIF4E3 plays an essential role in the asexual development of *M. oryzae*.

### MoeIF4E3 Is Required for the Full Virulence of *M. oryzae* to the Host Plant

**FIGURE 5:**
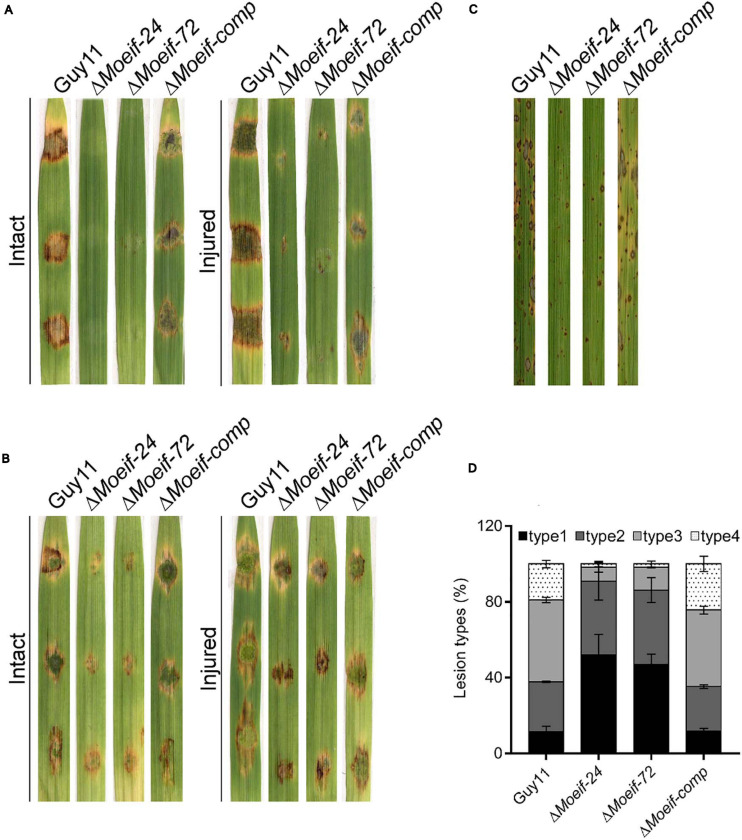
Contribution of MoeIF4E3 to promote the pathogenicity of *M. oryzae*. **(A)** Represent the hyphae mediated blast lesions on intact and injured barley leaves inoculated with mycelial plugs from Δ*MoeIF4E3* mutants (Δ*Moeif-24*, Δ*Moeif-72*), ΔMoeif-comp, and the wild-type strain. **(B)** Depicts the blast lesions due to spore inoculation of respective mutant, complemented strain, and Guy11 on intact and injured barley leaves. **(C)** Portrays disease lesions on 2 weeks old rice leaf from a susceptible rice cultivar CO39 after spraying with conidia suspensions of Guy11, Δ*MoeIF4E3* mutants, and the complemented strain. Photographs were taken after 6 days of inoculation. **(D)** A graph for scoring lesion types per 1.5 cm^2^ (1 to 4) from rice leaves inoculated with a spore suspension of Guy11, mutants, and complementation strains.

To evaluate the influence of MoeIF4E3 on the infectious development of the rice blast fungus during host-pathogen interaction, hyphae and spores obtained from Δ*Moeif4e3*, wild-type, and the complementation strain, were used to inoculate 8-days-old barley leaves as previously described (Li X. et al., 2017; Aliyu et al., 2019). Assessment of infection characteristics of the individual strains at 7-days post-inoculation (dpi) showed that the inactivation of *MoeIF4E3* severely compromised the penetration and virulence of the Δ*Moeif4e3* strains on intact and injured barley leaves (Figure 5A). Similar virulence defects were observed on barley and rice seedlings using independently inoculated spore suspensions prepared with asexual spores harvested from the Δ*Moeif4e3*, wild-type, and the complementation strains (Figures 5B,C). Further, comparative blast lesion index (Valent et al., 1991) assessment results showed that 50% of lesions recorded on leaf tissues of rice seedlings inoculated with the Δ*Moeif4e3* strains were type-1 (uniform dark brown lesion of about 0.5 mm diameter), and 40% were type-2 (small lesions with distinct centers, 1 mm). Lesions recorded on leaves of seedlings inoculated with the wild-type and the complementation strains were predominantly type-3 (medium size lesion with dark-gray centers and measuring about 2 mm in diameter) and type-4 (large size lesions whitish-gray centers and about 3–4 mm) (Figure 5D and Supplementary Figure 3). These observations suggest that MoeIF4E3 plays a vital role in the efficient colonization of host tissues and facilitates the establishment of the rice blast disease.

### MoeIF4E3 Is Also Required for Invasive Growth of *M. oryzae* Into Host Tissues

**FIGURE 6:**
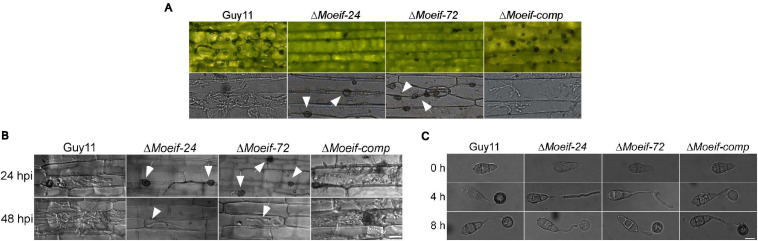
Contribution of MoeIF4E3 for invasive growth of *M. oryzae* in host tissues. **(A)** Development of appressorium-like structures and hyphal mediated penetration by the *MoeIF4E3* mutants and wild-type strain inoculated on barley leaves at 48hpi. Scale bar, 20 μm. **(B)** *In vivo* penetration and consequent sheath tissue- colonization activity was assessed using fluorescence microscopy at 24- and 48-hpi. **(C)** Germination and appressorium formation activity by conidia obtained from the respective mutants and the wild type on an artificial hydrophobic surface. Leaf-sheaths of susceptible rice cultivar CO39 were inoculated with conidia suspension of the same strains used for hyphal mediated penetration on barley leaves. Bar 40 μm.

Besides conidia, *M. oryzae* deploys hyphae initiating blast infection by forming tip appressorium-like structures on the tip of the germination hyphae (Kong et al., 2013; Lin et al., 2019). We assessed the impact of the *MoeIF4E3* gene deletion on the hyphae-mediated establishment of blast infection structures. The Δ*Moeif-24*, Δ*Moeif-72*, and wild-type strains were inoculated onto barley leaves. Microscopy examinations of the formation of appressorium-like structures, invasion efficiency of appressorium-like structures produced by the defective strains showed that targeted disruption of the *MoeIF4E3* gene compromised both the formation of appressorium-like structures and the leaf penetration capabilities of the Δ*Moeif*4e3 strains (Figure 6A). Similar penetration defects were recorded from additional histopathological assays conducted in this study by inoculating leaf sheaths of the susceptible CO39 rice cultivar with conidia suspension from the individual strains (Figure 6B). Furthermore, results obtained from evaluating the progression of appressorium morphogenesis for the *Moeif4e3*, and wild-type strains *in-vitro* revealed that the deletion of the *MoeIF4E3* gene delayed appressorium formation in the Δ*Moeif4e3* strains (Figure 6C). From these results, we speculate that MoeIF4E3 modulates the pathogenesis of the rice blast fungus through translational regulation of proteins required for appressorium formation, penetration, and colonization of host tissues.

### MoeIF4E3 Essentially Contributes to Different Stresses Response

**FIGURE 7:**
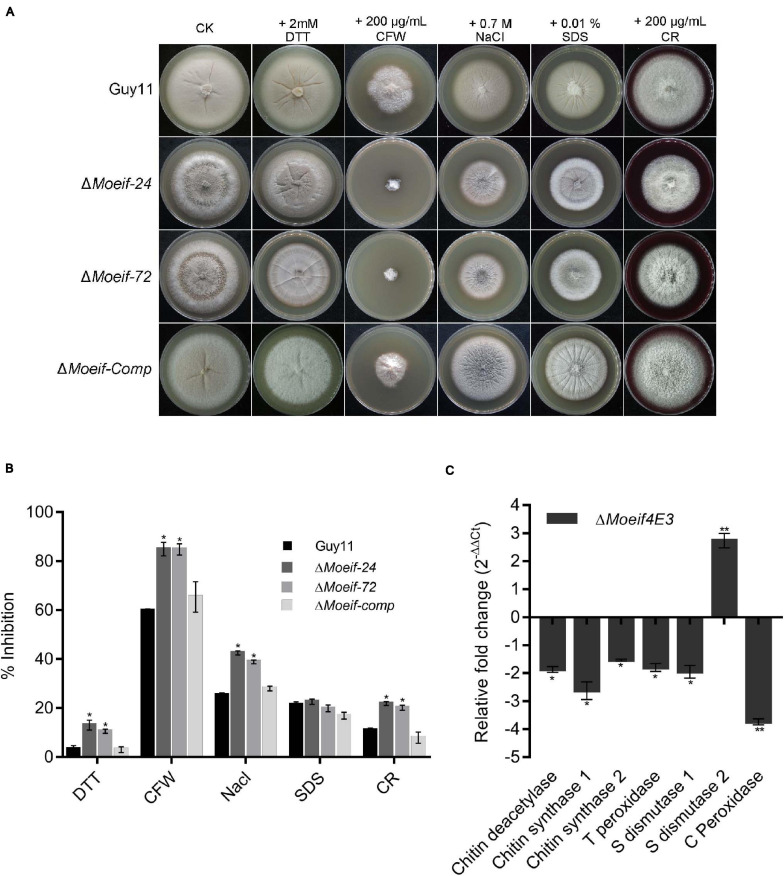
MoeIF4E3 deleted strains are sensitive to various stress in *M. oryzae*. **(A)** Colony morphology of Guy11 and *MoeIF4E3* deletion mutants on CM plates amended with different stress-inducing osmolytes. The colonies were photographed at 10 days post-inoculation. **(B)** Statistical analysis of growth inhibition rate of respective mutants and the wild-type strains under different stresses. The percent inhibition was obtained using this formula: Inhibition rate = (the diameter of untreated strain – the diameter of treated strain)/ (the diameter of untreated strain × 100%). Statistical evaluation of percent inhibition was done using non-parametric ANOVA, using the GraphPad Prism7 software. Error bars represent standard deviation from three replications. **(C)** Expression profiling of different cell-wall-related enzymes during vegetative growth of *MoeIF4E3* deleted mutant and Guy11 using qRT-PCR. Error bars represent standard deviation (SD). SD was calculated from three independent biological replications and three technical replicates, and significant levels were estimated using *t*-tests (*p < 0.05; **p < 0.01).

A large proportion of the transcriptional and translational regulatory mechanisms are activated in response to biotic and abiotic stress (Hinnebusch, 1994; Sheikh and Fornace, 1999; Ptushkina et al., 2004). The wild-type and D*Moeif* strains were cultured on CM supplemented with different stress-inducing agents, including 2 mM DTT (for reductive stress), 0.7 M NaCl (for osmotic stress), 0.2 mg/ml CFW (Calcofluor White interfering with chitin in the cell wall), 0.2 mg/ml CR (Congo Red, a cell wall stress and oxidative stress-inducing agents) for assessing cell wall integrity), and 0.01% SDS (sodium dodecyl sulfate, a surfactant for assessing cell membrane integrity) to determine the contribution of *MoeIF4E3* to attenuate stress responses in *M. oryzae*. Compared to the wild-type strain, the D*Moeif* strains displayed a higher sensitivity toward CFW and CR (Figures 7A,B). From these results, we infer that MoeIF4E3 mediated translational regulation directly or indirectly contributes to multiple stress responses in *M. oryzae.*

Furthermore, to unravel the influence *MoeIF4E3* on enzymes associated with cell wall and membrane integrity, we also performed qPCR assays to assess the transcription pattern of three chitin synthase-encoding genes (chitin deacetylase, chitin synthase1, chitin synthase2) and genes that code for enzymes that contribute to the enforcement of cell wall and cell membrane integrity through free radical scavenging. That included thioredoxin peroxidase, superoxide dismutase 1, superoxide dismutase 2, catalase-peroxidase in the wild-type, and the D*Moeif* strains. The comparative quantitative transcriptional analysis showed that targeted deletion of *MoeIF4E3* significantly down-regulated the expression of chitin deacetylase, chitin synthase 1, chitin synthase 2, thioredoxin peroxidase, superoxide dismutase 1, and catalase-peroxidase. On the contrary, the disruption of *MoeIF4E3* triggered a three-folds increase in the transcription level of superoxide dismutase 2 (Figure 7C). These results suggest *MoeIF4E3* contributes positively to cell wall integrity and cell wall stress homeostasis in *M. oryzae* via translational regulation of enzymes associated with cell wall biosynthesis and stress-inducing radicals.

### Targeted Replacement of *MoeIF4E3* Impacted Differently the Expression of Genes Coding for Proteins Associated With Translational Machinery of *M. oryzae*

**FIGURE 8:**
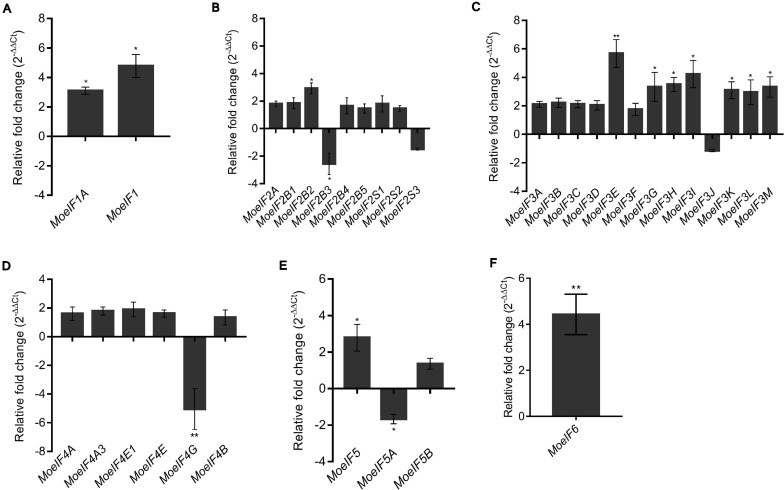
Relative fold change in the expression profile of translation initiation factors of *M. oryzae* in *MoeIF4E3* KO mutants. The graph shows expression profiling of **(A)**. eIF1 isomers, **(B)**. eIF2 complex, **(C)**. eIF3 complex, **(D)**. eIF4F complex and eIF4B, **(E)**. eIF5 complex, and **(F)**. eIF6 in vegetative hyphae of MoeIF4E3 KO mutants. Wild type Guy11 vegetative hyphae were used as control and assumed unity = 1. Error bars represent standard deviation (SD). SD was calculated from three independent biological replications along with three technical replicates. (**p < 0.01 and *p < 0.05, *t*-test).

To ascertain the impacts of *MoeIF4e3* gene deletion on the expression pattern of genes coding for proteins associated with individual eIF complexes in *M. oryzae*, we monitored the comparative expression pattern of eIF1, eIF2, eIF3, eIF4, and eIF5 complex coding genes in the *Moeif4e3* strains and the wild-type strain using RT-qPCR. We observed that targeted disruption of *MoeIF4e3* substantially downregulated the expression of all tested genes (Figures 8A–E). We also demonstrated that the targeted replacement of *MoeIF4e3* in the *Moeif4e3* strains caused a significant upregulation in the expression pattern of *MoeIF1*, and *MoeIF1A* (eIF1 complex), *MoeIF2B2* (eIF2 complex), *MoeIF3E, MoeIF3G, MoeIF3H*, *MoeIF3I, MoeIF3K, MoeIF3L*, and *MoeIF3M* (eIF3 complex), and *MoeIF6* (Figures 8A,C,E,F). These results suggest a synergetic coordination between *MoeIF4e3* and selected elements from other eIF complex. However, the exact conditions that drive exclusive coordination between subunits of eIF complexes identified in *M. oryzae* are not immediately obvious.

To assess expression pattern of genes coding for components of the MoeIF4F complex, including *MoeIF4A*, *MoeIF4A3, MoeIF4E1*, *MoeIF4E*, and *MoeIF4*G in the Δ*Moeif4e3* strain during pathogen-interaction, RNAs extracted from leaf sheath of 6-weeks old susceptible rice seedlings independently inoculated with spore suspensions prepared with asexual spores harvested from the Δ*Moeif4e3* and wild-type strains at 12-hpi (control stage), 24-, 36-, 72-, and 96-hpi, were used to conduct RT-qPCR analyses. The findings from these investigations revealed downregulation in the expression of *MoeIF4A*, *MoeIF4E*, and *MoeIF4G* (24-hpi), *MoeIF4E1*, and *MoeIF4E* (36-hpi) (Supplementary Figure 4). From these results, we inferred that MoeIF4E3 likely acts in association with other components of the eIF4 complex to promote the invasive development of the rice blast fungus during the early stages of pathogen-host interaction.

## Discussion

Rice blast fungus must undergo various cellular and developmental changes to infect the host plant and complete the disease cycle. These developmental changes are at the beginning of the *M. oryzae* infection cycle. Through extensive genetic studies over the past few decades, several genes have been identified to have critical roles (Yan and Talbot, 2016). Expression regulation of such genes either at the transcriptional or translational level allows an organism to respond to environmental changes. In previous studies, the eukaryotic translation initiation factor 4E (eIF4E), being a part of the pre-initiation protein complex (Culjkovic et al., 2007), has been associated with the translation of large proportions of mRNAs to protein in response to different signaling demands, including growth, reproduction, and stress tolerance (Inoki et al., 2005; Richter and Sonenberg, 2005). Eukaryotes possess multiple and varying isoforms of eIF4E; for example, three, four, five, and eight eIF4E isoforms were identified in humans, *Leishmania spp*., *A. thaliana*, and Drosophila, respectively (Hernández et al., 2005; Zinoviev et al., 2012; Frydryskova et al., 2016). We demonstrated that among the three eIF4E isoforms identified in rice blast fungus, isoform MoeIF4E3 displayed a significantly enhanced expression pattern during rice and *M. oryzae* interaction compared to isoforms MoeIF4E and MoeIF4E1. The differential expression pattern displayed by MoeIF4E isomers during host infection suggests individual isomers possibly influence the progression of cellular processes through independent regulation of different signaling pathways (Sonenberg and Gingras, 1998; Ruggero et al., 2004; Sun et al., 2005; Mamane et al., 2007). The primary occurrence of MoeIF4E3 in pathogen-rich fungal classes including *Leotiomycetes, Sordariomycetes*, and *Eurotiomycetes*, coupled with the close phylogenetic linage of MoeIF4E3 with orthologs in two thermo-dimorphic infectious fungal pathogens, *Sporothrix brasiliensis* and *Sporothrix schenckii* that causes the sporotrichosis, or “rose gardener’s disease” (a skin disease) in both humans and animals, especially in cats (Sanchotene et al., 2015; Almeida-Paes et al., 2016), is in line with our results that eIF4E3 likely contributes to the pathogenic development of filamentous fungus through translational regulation of proteins associated with pathogenesis.

The commencement of mRNAs’ translations of proteins occurs either on free-cytosolic ribosomes or ribosomes attached to the endoplasmic reticulum (rough-ER) (Reid and Nicchitta, 2015). Translation initiation factors facilitate the translation process by mediating the assemblage of ribosomes (large and small units), mRNA, and tRNA (“initiator”) to form the initiator complex (Pelletier and Sonenberg, 2019). Therefore, the localization of MoeIF4E3-GFP to the cytoplasmic region during physiological and pathological development of the rice blast fungus partly confirms its association with the cytoplasmic protein biosynthesis machinery. These observations are consistent with the localization pattern reported for MoeIF4E3 orthologs in *S. cerevisiae, S. pombe*, and mammals (Lang et al., 1994; Ptushkina et al., 2004; Willett et al., 2006). We accordingly concluded that eIF4E3 likely retains a conserved localization pattern across eukaryotes.

Protein requirement or expenditure of living organisms is generally defined by the individual organisms’ physiological and cellular needs at different developmental stages (Genuth and Barna, 2018). Also, while some set of proteins encoded by house-keeping genes are constitutively expressed at all developmental stages of the organism under normal conditions and are considered as proteins that are required for the maintenance of primary cellular function, other groups of proteins are selectively synthesized in response to the prevailing physiological and cellular status of the organism (Zhang and Li, 2004). Components of the initiation complex, including *eIF4E3* upstream transcriptional control pathways, essentially mediates the rapid and efficient translational regulation of a vast array of proteins required to support diverse developmental processes, including growth, cell proliferation, and reproduction (Hernández and Vazquez-Pianzola, 2005; Culjkovic et al., 2007). Here, we showed that the disruption of *MoeIF4E3* significantly attenuated vegetative growth and caused an approximate 70% reduction in the production of asexual spores. Earlier reports have equally implicated the eIF4E complex in regulating plant growth and cell differentiation in tobacco, and *S. cerevisiae*, respectively (Altmann et al., 1987; Combe et al., 2005). Meanwhile, the over-expression of *eIF4E* has been shown to trigger aberrant cell growth and malignant transformation (De Benedetti and Rhoads, 1990; Bianchini et al., 2008). These observations indicate that besides playing a conserved role in morphological development, *MoeIF4E3* assumes a unique role in promoting the asexual development of filamentous fungi.

Although studies have established a correlation between up-regulation in unique sets of genes coding for transcription factors, like proteins associated with signaling cascades, secretion, and trafficking pathways (Soanes et al., 2012; Cao et al., 2016; Oses-Ruiz et al., 2020), the commencement and progression of pathogenic development of the *M. oryzae*, is currently little known with regards to the influences of the translational regulation machinery on the pathogenic development of the rice blast fungus. Evidence obtained from this study showed that the disruption of *MoeIF4E3* severely compromised cell wall integrity and suppressed pathogenic development, invasion, and colonization efficiency of the rice blast fungus. These observations partly parallel the association of eIF4E1 with the progression of *Clover yellow vein virus* (ClYVV), *Chilli veinal mottle virus* (ChiVMV), and Pepper *veinal mottle virus* (PVMV) (Stein et al., 2005; Nieto et al., 2006; Ibiza et al., 2010; Wang and Krishnaswamy, 2012), coupled with the reported role of eIF4E2 in mediating stress tolerance in *S. pombe* (Sheikh and Fornace, 1999), suggesting that individual isoforms of the IF4E complex differentially influence the pathogenesis of infectious microbes possibly through translational regulation of pathogenicity or virulence proteins (factors) during the progression of host-pathogen interaction. Also, since the eIF4E hierarchically operates up-streams of transcriptional regulation of gene expression, it will be interesting to investigate how an individual component of the initiator complex coordinates with the transcriptional regulation machinery to regulate the physiological and pathological development of filamentous fungus.

In summary, functional genetics and biochemical characterization of targeted *MoeIF4E3* gene deletion strains generated in this study showed that MoeIF4E3 contributes positively to the enforcement of cell wall integrity and pathogenesis of the rice blast fungus, possibly by regulating the biosynthesis of enzymes and proteins required for stress homeostasis, pathogenic development and the virulence of *Magnaporthe oryzae.* This study has provided an insight into the significance of the eIF4E in the pathogenesis of filamentous fungi. These findings underscore the need to comprehensively evaluate the contributions of the subsequent isoforms and components of the translation initiator complex on the progression of host-pathogen interaction and their potential exploration in sustainable development anti-blast compounds.

## Materials and Methods

### Domain Architecture of eIF4E3 and Its Phylogenetic Analysis

Amino acid sequences of all the eIF4E identified in different fungal species were acquired from a publicly available NCBI protein database for domain prediction. The domain prediction was performed using Pfam domain prediction tool^3^, and later domain base phylogenetic analyses of 20 eIF4E were carried out using amino acid sequences of defined eIF4E domain regions. Domain sequences were first aligned using the online multiple sequence alignment tool^4^. The aligned sequence was then used to generate the phylogenetic tree phylogeny in MEGA 7.0 using the maximum-likelihood method based on the Poison correction model. The evolutionary history of each branch of the tree was inferred from 1000 bootstrap replicates. Branches corresponding to partitions reproduced in less than 50% bootstrap replicates were collapsed. Domains showing more recent lineage are presented with the same color.

### Fungal Isolate and Culture Conditions

*Magnaporthe oryzae* strain (Guy11) used in this study for generating MoeIF4E3 knockout mutants was kindly given by Dr. Didier Tharreau (CIRAD, Montpellier, France). Competent cells used for the multiplication of constructed vectors were prepared from *E. coli* strain DH5α. The wild type Guy11, MoeIF4E3 mutants, and the respective complementation strains were cultured on sterilized CM (complete growth medium) containing 0.6% casamino acid, 0.6% yeast extract, 1% sucrose, and 2% agar at 28°C (Chen et al., 2008). For growth assay, Minimal media (MM, containing 50 ml of 20× nitrate salts, 1 ml of 1,000× trace elements, 1 ml of 1,000× vitamin solution, 1% glucose, 2% agarose) with pH 6.5 was used. The DH5α strain, used to generate competent cells, was cultured in sterilized LB (lysogeny broth, pH: 7.0) medium containing 1% tryptone, 1% NaCl, and 0.5% yeast extract. For protoplast preparation, RNA isolation, and gDNA extraction, individual strains were cultured in liquid CM and incubated in a rotatory shaker with a speed of 110 rpm under 28°C for 3–4 days.

For sporulation assay, wild type strain and mutants and their complementation strains were inoculated on RBM (rice bran medium, pH: 6.0–6.5), which was prepared using 4% rice bran and 1.5% agar. The plates were incubated at 28°C for 7–10 days with 12 h of photoperiod before scratching the vegetative hyphae and were again incubated in light for 3 days (Lin et al., 2019). The colony diameter of each strain was measured after 10 days of growth on CM plates supplemented with different stress-inducing elements (2 Mm DTT, 200 μg/mL Calcofluor white (CFW), 0.7M NaCl, 0.01% SDS, 200 μg/mL Congo red) to investigate the stress response of the MoeIF4E3 deletion mutants.

### Protoplast Preparation

For transformation, protoplasts were prepared following standard protocols (Abdul et al., 2018). The wild type strain was first cultured in liquid CM medium and placed in a 28°C shaker for 3–4 days at 110 rpm, and after that, the media was drained off, and the cultured colonies were ground using mortar and pestle, then resuspended in fresh liquid CM and incubated for 12–14 h in a 28°C shaker at 130 rpm. Afterward, the Guy11 mycelium was filtered from liquid medium and washed twice with sterilized ddH_2_O and 1M sorbitol. Washed mycelium was dried on filter paper and then resuspended in a conical flask containing 40 ml of lysing enzyme solution (conc:1g lysing enzyme/100 ml of 1M sorbitol) and incubated in a rotatory shaker at 30.5°C for 2.5–3 h. Protoplasts were filtered from the lysing solution using a double layer of Mira cloth and centrifuged at 4°C for 10 min at 5,000 rpm. The supernatant was removed; the pellet was gently resuspended in 1 M sorbitol STC, and again centrifuged at 4°C for 10 min at 5,000 rpm. Finally, the pellet was decoagulate in 1ml STC, and protoplast concentration was estimated by microscopy. DMSO (7%) was added into the final volume of protoplasts (adjusted conc.1 × 106), and 300 μl volume was aliquoted into 2 ml sterilized tubes. Tubes containing protoplast were stored at −80°C for further use.

### Generation of MoeIF4E3 Knockout Mutants and Complementation Strains

Knockout vectors used for deleting the *MoeIF4E3* gene in *M. oryzae* were constructed using a split marker approach. For it, 0.8 kb upstream and 0.9 kb downstream regions were amplified using MoeIFAF/AR and MoeIFBF/BR primers pairs, respectively (Supplementary Table 3). The resulting PCR products were ligated with a hph cassette fragment amplified with primers HYG/F+HY/R and YG/R+HYG/R (Aliyu et al., 2019) by overlapping PCR. For transformation, targeted gene deletion using a homologous recombination approach was used as described (Zhang et al., 2016). Potential transformants were prescreened with a PCR assay using gene-specific ORF and UAH primer pairs (Supplementary Table 3) and later confirmed with qPCR (Supplementary Figure 1) and Southern blotting (Figure 2). The MoeIF4E-GFP fusion vector was constructed by amplifying a 4 kb fragment containing complete *MoeIF4E3* ORF along with the native promoter region using MoeIFcompF/compR primer pair as described (Lin et al., 2019). Constructed vectors were transformed into the MoeIF4E deletion mutant using Guy11 protoplast for generating the *MoeIF4e3* complementation and GFP fusion strains, respectively. Neomycin (G418) resistant transformants were screened with respective ORF primers and observed under a microscope for GFP signals.

### *Magnaporthe oryzae* Genomic DNA, RNA Isolation, and qPCR

Genomic DNA of wild type strain Guy11, ΔMo*eif-24*, ΔMo*eif-72*, and complementation strains were extracted using the SDS-CTAB method. Strains were first cultured in liquid CM medium at 28°C for 3–4 days in a shaker at 100 rpm. The colonies were filtered, washed with sterilized ddH2O, and dried completely using filter papers. After drying, colonies were frozen in liquid nitrogen and ground into a very fine powder using mortar and pestle. Around 0.1g of ground mycelia was resuspended in 1 ml of DNA extraction buffer (150 mM NaCl, 30 μg/mL proteinase K, 10 mM Tris–HCl, pH7.4, 50 mM EDTA), and was vortex to homogenize the mixture, then incubated at 37°C for 1 h in a water bath after adding 100 μL SDS (20%). After removing the sample from the water bath, 150 μl NaCl (5M) and one-tenth volume of 10% CTAB+0.75M NaCl mixture were added into the solution and then placed in a water bath at 65°C for 30 min. After 30 min, 400 μl phenol-isoamyl alcohol (1:1) was added and thoroughly mixed before centrifuging at 12,000 rpm for 15 min. The supernatant was pipetted into new, 2 ml, sterilized tubes containing an equal volume of chloroform. After shaking heavily, the mixture was centrifuged for 10 min at 12,000 rpm. The supernatant was transferred into new Eppendorf tubes containing two-fold volumes 100% ethanol. The mixture was shaken thoroughly and kept at −20°C for 1 h or overnight. After centrifuging for 15 min at 12,000 rpm, the supernatant was discarded, and tubes containing a DNA pellet were let to dry. A 500 μl of sterilized ddH_2_O and 2 μl RNAse for RNA degradation was added and the tubes kept in a 37°C water bath for 30 min. The samples were then centrifuged at 15,000 rpm for 10 min after adding chloroform isoamyl alcohol (24:1). The supernatant was transferred into new sterilized Eppendorf tubes, and an equal volume of chloroform was added. The tube was thoroughly shaken before centrifuging for 10 min at 12,000 rpm. The supernatant was pipetted into new EP tubes containing two folds of absolute ethanol. After keeping for 2 h at −20°C, the mixture was centrifuged and washed twice with 70% ethanol and dried before adding ddH_2_O. The purified DNA was used later for southern Blot.

For RNA extraction Guy11, ΔMo*eif-24*, ΔMo*eif-72*, and the complementation strain were harvested in liquid CM as described above. After 3 days, RNA was extracted from each strain using Magen universal RNA kit following the method described (Aliyu et al., 2019) and was subjected to reverse transcription using SYBR Premix Ex. Taq (Tli RNaseH Plus) (Takara Biomedical Technology, Beijing Co., Ltd) for the preparation of cDNA. Real-time RT-PCR (RT-qPCR) was performed to detect the expression level of MoeIF4E3 in wild type, and MoeIF4E3 deleted strains following the standard protocol as described (Abdul et al., 2018). The tubulin was used as positive control and reference gene. Consistent values were obtained from three independent biological replications and three technical replicates for each independent experiment.

### Virulence Test, Appressorium Formation, and Infection Penetration Assays

To examine the virulence of Guy11, *MoeIF4E3* deleted mutants and their complementation strains, conidia collected from 10 days old RBM plates were diluted to a concentration of 5 × 10^4^ conidia/ml in 0.02%v/v tween-20. Conidial suspension of each strain was used to spray the three-leaf stage of CO39 rice seedlings. These were then kept in the dark chamber for 24 h at 28°C provided with 90% humidity before being transferred to a humid chamber provided with a 12 h light/12 h dark photoperiod. The disease lesions were observed on 7-dpi. Types of lesions were counted per 1.5 cm^2^ rice leave surface area using the lesion scoring method (Valent et al., 1991) and were presented in a graphical method using Prism 5. For infection assays with mycelia, fungal blocks of the same size were inoculated on 7 days old intact and injured barley leaves and were provided with the same conditions as for the rice infection assay. For infection assays with conidia suspension on barley leaves, 30 μl conidia suspension containing 3–5 conidia from wild type, *MoEif4E3* deleted mutants, and complementation strains were prepared individually. These droplets were inoculated on intact and injured barley leaves and were incubated under similar conditions as described above.

For appressorium formation, 20 μl droplets of the conidial suspension were placed on Fisher Scientific (St Louis, MO, United States) hydrophobic plastic coverslips and incubated at 25° under humid conditions in the dark (Li Y. et al., 2017). At 4hrs intervals, appressorium development was monitored under an optical microscope. For infection penetration assay, rice sheath epidermal cells of 4 weeks old CO39 rice cultivar were inoculated with a conidia suspension containing 2.0 × 10^5^ conidia/mL and incubated at 28°C under humid conditions. The infection penetration efficacies of each strain were observed at 24 and 48 hpi.

Fungal blocks were cultured in liquid CM and were inoculated on barley leaves before incubating under the dark and humid conditions at 26°C to observe the appressorium-like structure formation efficacies of individual strains. At 24 and 48 hpi, the formation of appressorium-like structures was monitored under an optical microscope. For monitoring the host colonization in barley epidermal cells, fungal strains cultured in liquid CM were inoculated on the backside of the barley leaves and were provided with similar conditions as described above. Fungal penetration was monitored at 48 and 56 hpi.

### Microscopy Assay

For observing conidia germination, appressorium formation, and infection penetration, a Nikon TiE system (Nikon, Japan) was used. For confocal examination, the observation was carried out as previously described (Wang et al., 2011).

## Data Availability Statement

The original contributions presented in the study are included in the article/Supplementary Material, further inquiries can be directed to the corresponding authors.

## Author Contributions

JN, ZW, QC, and WB conceived and design the research. JN and ZW acquired funding for the research. JN, WB, AS, LL, XC, QA, XH, SP, and SC prepared and processed the experimental samples and carried out the experiments. JN, ZW, and QC prepared the manuscript. All authors contributed to the article and approved the submitted version.

## Conflict of Interest

The authors declare that the research was conducted in the absence of any commercial or financial relationships that could be construed as a potential conflict of interest.

## Footnotes

1 http://fungidb.org/fungidb/

2 https://www.kegg.jp/kegg-bin/show_organism?org=mgr

3 http://pfam.xfam.org/search/sequence

4 https://www.ebi.ac.uk/Tools/msa/clustalo
