# Supplementary material for: Translation Initiation Factor eIF4E Positively Modulates Conidiogenesis, Appressorium Formation, Host Invasion and Stress Homeostasis in the Filamentous Fungi Magnaporthe oryzae
Source: Front Plant Sci. 2021 Jun 16;12:646343. doi: 10.3389/fpls.2021.646343 (PMC8244596; doi:10.3389/fpls.2021.646343)
Supplement: Supplementary file 1 [file Data_Sheet_1.pdf]

<u>List of Supplementary Figures and Tables</u>

Table S1. List of fungal species from different taxonomic class containing MoeIF4E3 orthologous

**Group**	**Class**	**Organism**
Oomycetes	*Not found*	
**Fungi**	*Agaricomycetes*	*Not found*
	Blastocladiomycetes	*Not found*
	Pneumocystidomycetes	*Not found*
	Pucciniomycetes	*Not found*
	Saccharomycetes	*Not found*
	Schizosaccharomycetes	*Not found*
	Tremellomycetes	*Not found*
	Ustilaginomycetes	*Not found*
	Zygomycetes	*Not found*
	Glomeromycetes	*Not found*
	Chytridiomycetes	*Cenococcum geophilum*
	Eurotiomycetes	*Aspergillus fumigatus*
	Eurotiomycetes	*Penicilliopsis zonata*
	Eurotiomycetes	*Aspergillus brasiliensis*
	Eurotiomycetes	*Aspergillus clavatus*
	Eurotiomycetes	*Aspergillus luchuensis*
	Eurotiomycetes	*Aspergillus novofumigatus*
	Eurotiomycetes	*Aspergillus ochraceoroseus*
	Eurotiomycetes	*Aspergillus tubingensis*
	Eurotiomycetes	*Aspergillus zonatus*
	Eurotiomycetes	*Cladophialophora carrionii*
	Eurotiomycetes	*ladophialophora immunda*
	Eurotiomycetes	*Histoplasma capsulatum*
	Eurotiomycetes	*Paracoccidioides brasiliensis*
	Eurotiomycetes	*Paracoccidioides lutzii*
	Eurotiomycetes	*Talaromyces marneffei*
	Eurotiomycetes	*Talaromyces stipitatus*
	Eurotiomycetes	*Uncinocarpus reesii*
	Leotiomycetes	*Botrytis cinerea*
	Leotiomycetes	*Sclerotinia sclerotiorum*
	Sordariomycetes	*Fusarium fujikuroi*
	Sordariomycetes	*Fusarium graminearum*
	Sordariomycetes	*Fusarium oxysporum*
	Sordariomycetes	*Fusarium proliferatum*
	Sordariomycetes	*Fusarium verticillioides*
	Sordariomycetes	*Lomentospora prolificans*

	Sordariomycetes	*Magnaporthe oryzae*
	Sordariomycetes	*Neurospora crassa*
	Sordariomycetes	*Neurospora tetrasperma*
	Sordariomycetes	*Scedosporium apiospermum*
	Sordariomycetes	*Sordaria macrospora*
	Sordariomycetes	*Sporothrix brasiliensis*
	Sordariomycetes	*Sporothrix schenckii*
	Sordariomycetes	*Trichoderma reesei*

List of MoeIF4E3 orthologous in *M. oryzae*

CLASS	ORGANISM	GENE ID	Given name	%protein-query covered	domain %-query covered	protein length	domain ranged
Sordariomycetes	*M. oryzae*	MGG_08170	MoeIF4E3	100%-100	100%-100	368 aa	152-309
Sordariomycetes	*M. oryzae*	MGG_12301	MoeIF4E	28.93%-95.1	96%-31.65	271aa	48-209
Sordariomycetes	*M. oryzae*	MGG_04647	MoeIF4E1	36.11%-50	86%-35.92	244aa	42-224

Table S2. List of MoeIF4E3 orthologous used for sequence homology

	CLASS	ORGANISM	GENE ID	Given name	%protein-query covered	domain %-query covered	protein length
1	Eurotiomycetes	*Aspergillus fumigatus*	AFUB_051690	AfeIF4E3	52.89%-89	66.24%-99	347aa
2	*Eurotiomycetes*	*Penicilliopsis zonata*	ASPZODRAFT_129280	PzeIF4E	54.34%-94	67.09%-100	373aa
3	*Eurotiomycetes*	*Aspergillus brasiliensis*	ASPBRDRAFT_76906	AbeIF4E	51.96%-89	64.56%-100	350aa
4	*Leotiomycetes*	*Botrytis cinerea*	Bcin15g02340	BceIF4E	57.44%-89	70.06%-99	339aa
5	*Leotiomycetes*	*Sclerotinia sclerotiorum*	SS1G_07827	SseIF4E	55.24%-94	70.06%-99	342aa
6	*Sordariomycetes*	*Fusarium fujikuroi*	FFUJ_03080	FfeIF4E	59.17%-91	71.34%-99	341 aa
7	*Sordariomycetes*	*Fusarium graminearum*	FGRAMPH1_01T23195	FgeIF4E	57.14%-98	70.25%-100	338 aa
8	*Sordariomycetes*	*Fusarium oxysporum*	FOYG_02867	FoeIF4E	58.88%-91	70.70%-99	341 aa
9	*Sordariomycetes*	*Fusarium proliferatum*	FPRO_04851	FpeIF4E	59.17%-91	70.70%-99	341 aa
10	*Sordariomycetes*	*Fusarium verticillioides*	FVEG_05198	FveIF4E	58.88%-91	71.34%-99	341aa
11	*Sordariomycetes*	*Lomentospora prolificans*	jhhlp_006576	LpeIF4E	52.99%-98	68.35%-100	386aa
12	*Sordariomycetes*	*Magnaporthe oryzae*	MGG_08170	MoeIF4E3	100%-100	100%-100	368 aa
13	*Sordariomycetes*	*Neurospora crassa*	NCU09546	NceIF4E3	63.54%-92	75.80%-99	355aa
14	*Sordariomycetes*	*Neurospora tetrasperma*	NEUTE1DRAFT_75712	NteIF4E	63.54%-92	75.16%-99	355aa
15	*Sordariomycetes*	*Scedosporium apiospermum*	SAPIO_CDS8916	SaeIF4E4	51.34%-97	67.72%-100	354 aa
16	*Sordariomycetes*	*Sordaria macrospora*	SMAC_07065	SmeIF4E3	64.07%-92	75.16%-99	357aa
17	*Sordariomycetes*	*Sporothrix brasiliensis*	SPBR_07228	SbeIF4E3	62.29%-90	80.89%-99	422aa
18	*Sordariomycetes*	*Sporothrix schenckii*	SPSK_05268	SsceIF4E3	62.57%-90	80.89%-99	422aa
19	*Sordariomycete s*	*Trichoderma reesei*	TRIREDRAFT_103322	TreIF4E3	56.82%-93	71.34%-99	337aa
20	Sordariomycetes	Trichoderma virens	TRIVIDRAFT_33839	TveIF4E3	57.26%-93	71.34%-99	337aa
21	Saccharomyces	Saccharomyces cerevisiae	NP_014502	SceIF4E	34%-44	33%-99	213 aa
22	Planta	Arabidopsis thaliana	NP_193538	AteIF4E3	35%-43	34%-88	235 aa
23	Animalia	Drosophila melanogaster	NP_648194	DmeIF4E3	35%-45	38%-93	244aa
24	Humans	Homo Sapiens	NP_001124150	HseIF4E	37%-44	36%-96	237 aa

Table S3. List of primers used in this study

Primer Name	Primer sequence(5'-3')
eIF AF	GGCTCTCGGGATGATGG
eIF AR	TTGGTAGGGCGGCTGGT
eIF OF	CACCAACCACCACCCTTCA
eIF OR	TCAGCACCGCACCACAGACC
eIF BF	TGTTTTGTGGTGGGATGG
eIF BR	AAAGGGAAGGCTGCGTAG
eIF UAH F	GGGAACATCCATCCTCAA
eIF UAH R	GCTCCATACAAGCCAACC
eIF Comp F	TCGTCGGGAATGGCGTA
eIF Comp R	TTGAGTCGCGCCGCTG
eIF4E3 qPCR F	GGAATGGTGAGGATATTTTG
eIF4E3 qPCR R	ATTTTGTTTACGCTTCTCC
eIF4E qPCR F：	GAAGTTGGTGCAGCTTGTTG
eIF4E qPCR R：	AGTTGTCGGGCTTGGGTC
eIF4E1 qPCR F：	ATTCCCATCTCTCCCGAT
Eif4E1 qPCR R：	TCCTCTACTGAGTTAAATGTGATA
ChitinD qPCR F	CCGTGCTAAAGGTCTCTC
ChitinD qPCR R	TTAGTTGCAAGTGCCAAAG
ChitinS1 qPCR F	TTATTCTACTCGCCTTGA
ChitinS1 qPCR R	CTTCATTACTGTCGGATC
ChitinS2 qPCR F	TACACTCTGATCCTACTC
ChitinS2 qPCR R	AACTGCCTTATGACAATG
T peroxidase qPCR F	GAGTGTTCGTTATTGACAAGA
T peroxidase qPCR R	CTCCTCTACCACCTCCTTA
C peroxidase qPCR F	CTCCTTCCAAGCTTATCCA
C peroxidase qPCR R	CCTTCTTGCCACTCTTCT
S dismutase 1 qPCR F	CTGGAAAAGACCGAAAAGG
S dismutase 1 qPCR R	CCACCACCTGCTAACATA
S dismutase 2 qPCR F	CGTATTTGGCGTTGACAT
S dismutase 2 qPCR R	ACAGGGAAGCATGTAGTG
eIF1A qPCR F	GTCACTGAGAGGCATCTTC
eIF1A qPCR R	CTTGTGTCGCTCGTTAGG
eIF1 qPCR F	CGAGGACACTGGTGATAA
eIF1 qPCR R	CTTCTTTGGGATGCCTTG
eIF2S1 qPCR F	TATATCAGCAAGCGGTTGA
eIF2S1 qPCR R	TACTGGTAAGAACGTAGAGC
eIF2S2 qPCR F	AGTATCTGTTTGCTGAGTTG
eIF2S2 qPCR R	CAGTCTTGATGGCAGTAAC

**eIF2S3 qPCR F**	TCAGCAGAATAGTGTCCTT
**eIF2S3 qPCR R**	GGAGCAGGTAGAAGTTGA
**eIF2A qPCR F**	AACCGTCTGGCTAACATT
**eIF2A qPCR R**	CTTCTTCATCTGAGTATCTTCG
**eIF2B1 qPCR F**	TCTACTTTGCTGCCGAAA
**eIF2B1 qPCR R**	ATCATCTTTGGCGGAGTAT
**eIF2B2 qPCR F**	CGACGGTTGAGAAGTTTC
**eIF2B2 qPCR R**	ATCTTGTGGCTTGCTAGT
**eIF2B3 qPCR F**	CCATCAAGGAGTCTGTCA
**eIF2B3 qPCR R**	ACTTCGCATTCCATCAAC
**eIF2B4 qPCR F**	AGGCGTCAAGGTTTCATA
**eIF2B4 qPCR R**	CGTTGTAGGTCAGAGGTT
**eIF2B5 qPCR F**	AATGAAGACAGCAGTAGGAA
**eIF2B5-qPCR R**	TTAATTGTACGCCGAATGAG
**eIF5-qPCR F**	AGGAGTTTGGCATTGAGA
**eIF5-qPCR R**	GGTGGTAGTACAGTTGAAGA
**eIF5A-qPCR F**	AGAGATGAGGGAGGACATTA
**eIF5A-qPCR R**	GGAACCTCCTCACCCATA
**eIF5B-qPCR F**	CATCAAGACCAACATCAGAG
**eIF5B-qPCR R**	TCCAAGTCAGATTCGTCAT
**eIF6-qPCR F**	CTCAGTTCGAAAACTCCAA
**eIF6-qPCR R**	TGTAAGACGACCCACTATC
**eIF4A-qPCR F**	CTTATTGCCACCGACTTG
**eIF4A-qPCR R**	TGCTGTAGAACTGCTCAA
**eIF4A3-qPCR F**	ACAGCCGAGTGTTGATAT
**eIF4A3-qPCR R**	TCGATGTCTCTGAGGATTC
**eIF4B-qPCR F**	GAGTGGTAACTGGAGGTC
**eIF4B-qPCR R**	CTAGGATGCAATAGGACGA
**eIF4E1-qPCR F**	ATTCCCATCTCTCCCGAT
**eIF4E1-qPCR R**	TCCTCTACTGAGTTAAATGTGATA
**eIF4E3-qPCR F**	CAAGATTGAGTGGAAGAGC
**eIF4E3-qPCR R**	CTGCTGTTTCTGGTCATC
**eIF4G-qPCR F**	CATCAGCATCAAGGACAAG
**eIF4G-qPCR R**	TGTATAACTCACCAATGAACTG
**eIF3K-qPCR F**	CGCTTCTGGGCCACTATC
**eIF3K-qPCR R**	TTCATCACGGAGGCTTGC
**eIF3A-qPCR F**	ACAGGGTCCCATCTTCAA
**eIF3A-qPCR R**	CAGCACGAGCAAGGTTAA
**eIF3B-qPCR F**	GAAGTCTCAGGCAACAAC
**eIF3B-qPCR R**	TCGTAGGTGCTTGAAGTT
**eIF3C-qPCR F**	TACCACGAGAGGCAAATC

**eIF3C-qPCR R**	GCGTCTCAATGCTCAATG
**eIF3D-qPCR F**	TAAGGCAGATGTCCTCAAG
**eIF3D-qPCR R**	CGCTTCAGACACATATCG
**eIF3E-qPCR F**	TCGCATCTGAGATTCTGT
**eIF3E-qPCR R**	GAGGTCTGGATAGTGTTGA
**eIF3F-qPCR F**	TGTCAATACTAGACCACGC
**eIF3F-qPCR R**	TCATGGCTGGTGGTGTAC
**eIF3H-qPCR F**	AACCACCATTCCTACATCTC
**eIF3H-qPCR R**	TTGCCACGCTGTAATCTT
**eIF3I-qPCR F**	GAGGCTAGGTTCTACCATAA
**eIF3I-qPCR R**	CTGCTCCATCTTGTTCTTC
**eIF3L-qPCR F**	GCACTTCACAACCTACTACTA
**eIF3L-qPCR R**	CACCGAACTTCTCACGAA
**eIF3M-qPCR F**	AGGAGAACCTTGACAATGA
**eIF3M-qPCR R**	CCGAGTGAATCAGGAAGA

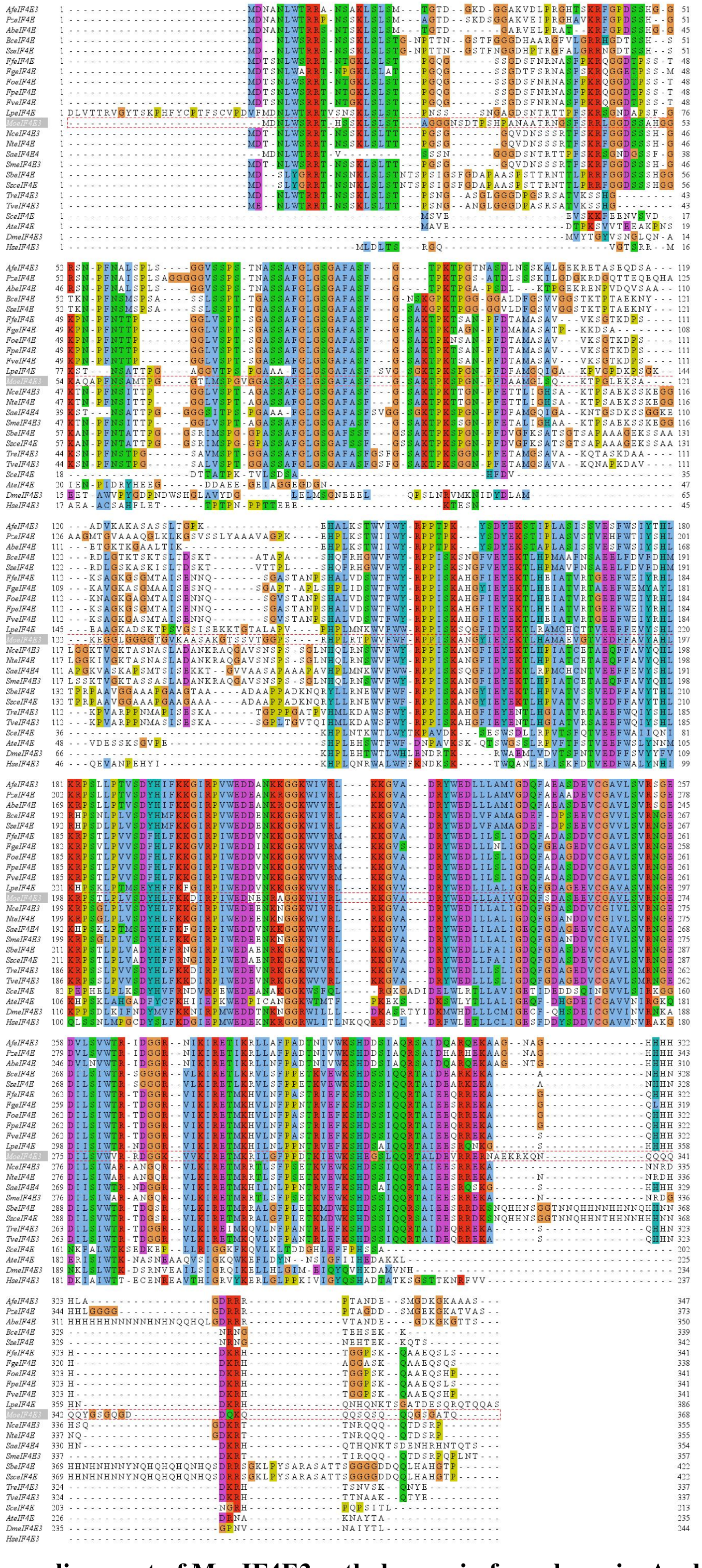

Fig. S1. Multiple sequence alignment of MoeIF4E3 orthologous in fungal specie, Arabidopsis, Drosophila and Humans.

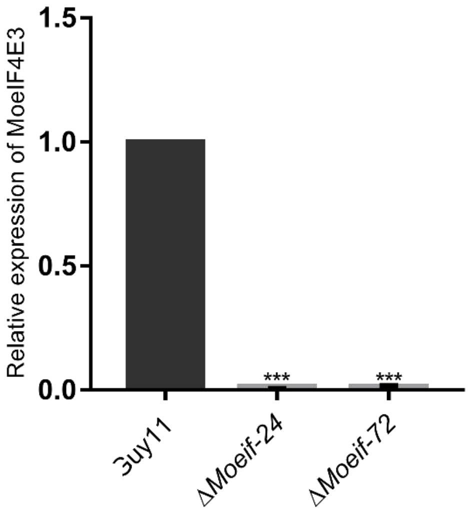

Fig. S2. Relative expression of MoeIF4E3 in wild type and MoeIF4E KO mutants. MoeIF4E3 Knockout mutants were confirmed through expression profiling of MoeIF4E3 in each strain as compared to wild type with qPCR. Results were generated from three independent biological with five technical replicates.

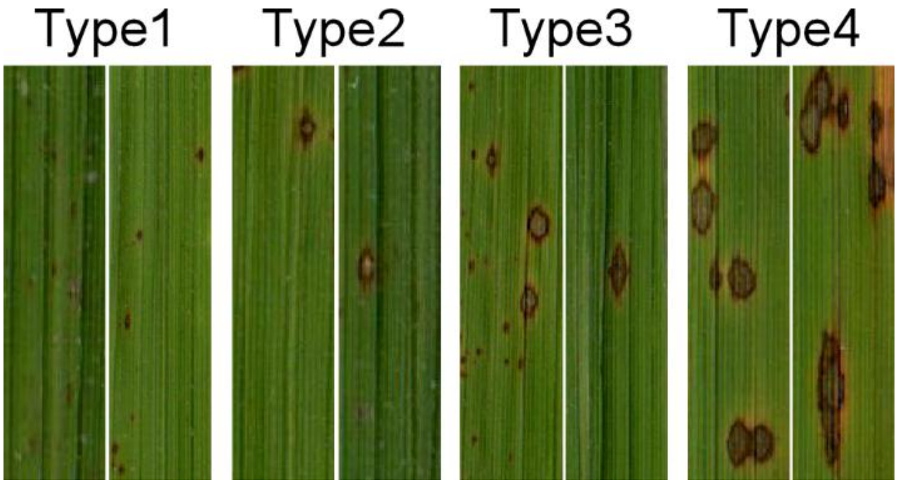

Fig. S3. Different types of lesion on leaf segments of rice cultivar C039. Type 1: uniform dark brown spot (0.5mm in diameter), Type 2: small lesions with distinct centers (1 mm in diameter), Type 3: small eyespot lesions (2 mm in length) with tan centers surrounded by dark brown margins, Type 4: large size eyespot lesions (3-4 mm in length).

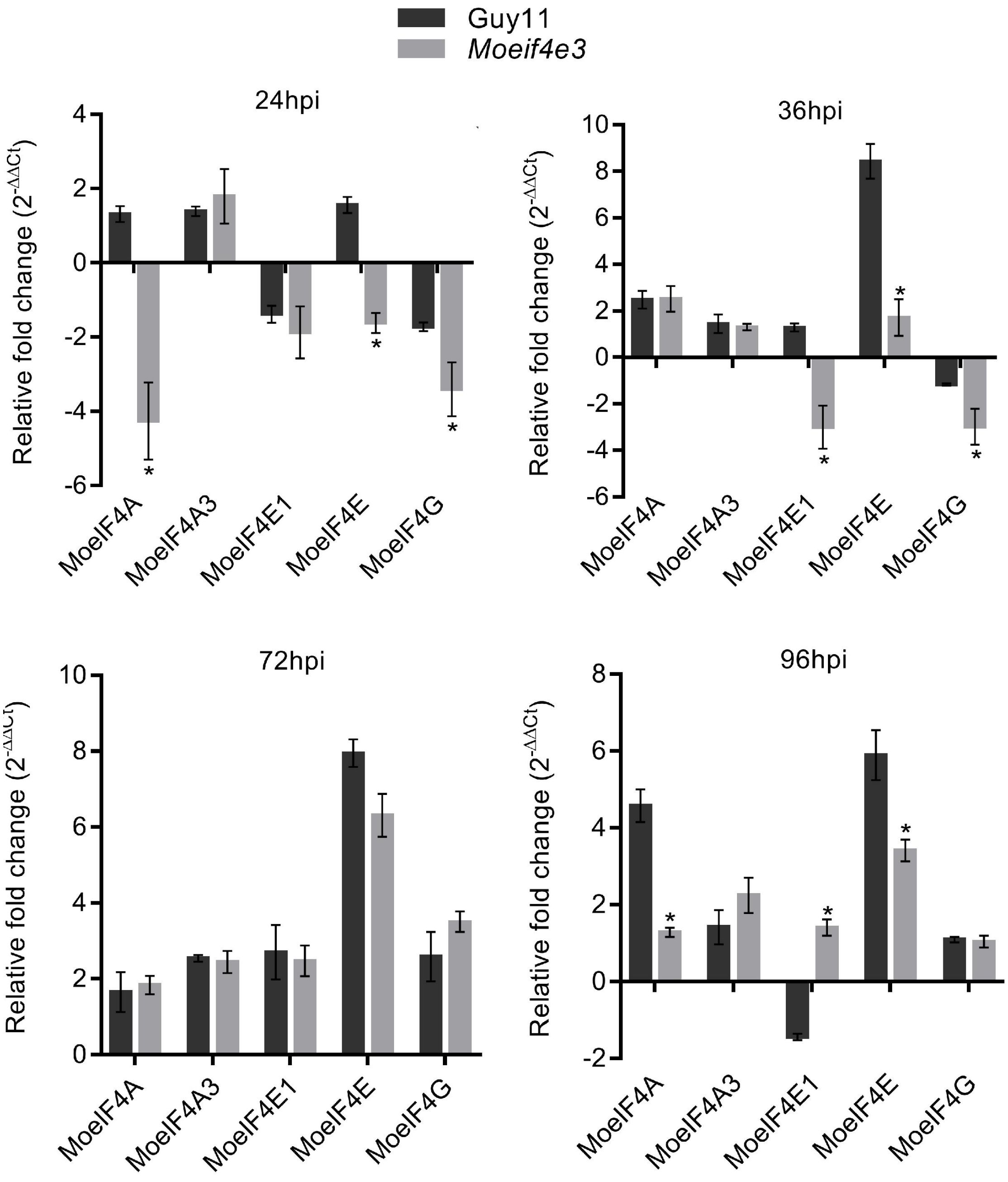

Fig. S4. Transcription expression pattern of MoeIF4F complex, during host pathogen interaction. The relative expression level of MoeIF4F complex proteins i.e. MoeIF4A, MoeIF4A3, MoeIF4E1, MoeIF4E and MoeIF4G in Wild type Guy11 and Moeif4e3 deletion strains at different time intervals i.e. 12 hpi, 24hpi,36hpi,72hpi and 96hpi was assessed using qPCR. Expression at 12hpi was used as a control stage and was assumed as unity (the expression level of MoeIF4A, MoeIF4A3, MoeIF4E1, MoeIF4E and MoeIF4G at 12 hpi =1). Error bars represent standard deviation (SD). SD was calculated from three independent biological replications along with three technical replicates. (*, $P < 0.05$ by t-test).
